# A first step towards computational design of W-containing self-healing ferritic creep resistant steels

**DOI:** 10.1080/14686996.2020.1814679

**Published:** 2020-09-14

**Authors:** Hao Yu, Wei Xu, Sybrand van der Zwaag

**Affiliations:** a State Key Laboratory of Rolling and Automation, Northeastern University, Shenyang, China; b Novel Aerospace Materials Group, Faculty of Aerospace Engineering, Delft University of Technology, Delft, The Netherlands

**Keywords:** Self-healing properties, heat resistant steels, alloy design, thermodynamics and kinetics, laves phase nucleation barrier, 106 Metallic materials, 407 CALPHAD / Phase field methods

## Abstract

In this work, we combine a generic alloy-by-design model with a novel concept, the nucleation barrier for the formation of Laves phase to fill the creep cavities, in order to develop multi-component creep resistant steels with kinetically tuned self-healing behaviour. In the model the high-temperature long-term strength is estimated by integrating precipitation strengthening due to M23C6 carbides and solid solution strengthening, while the optimized compositional solutions are determined by employing the coupled thermodynamic and kinetic principles. W-containing Laves phase herein is selected as the self-healing agent to autonomously fill the grain boundary cavities, so as to prolong the creep lifetime. To achieve the effective healing reaction, the nucleation time for Laves precipitates are expected to coincide simultaneously with which creep cavities start to form or reach a healable size. Using experimental data from literature, an empirical relationship to estimate the incubation time for Laves phase formation has been constructed, from which the thermodynamic driving force for onset of precipitation as a function of temperature and intended precipitate nucleation time was derived. Three sample alloys have been selected among the desirable solutions, which are predicted to have the same strength but widely different Laves phase nucleation times. The calculations are also performed for different use temperatures to explore the compatibility between high temperature strength and timely cavity filling behaviour. In its current form the model is not expected to yield the truly optimal composition but to demonstrate how the kinetics of the healing reaction can affect the predicted optimal alloy compositions.

## Graphical abstract

**Figure:**
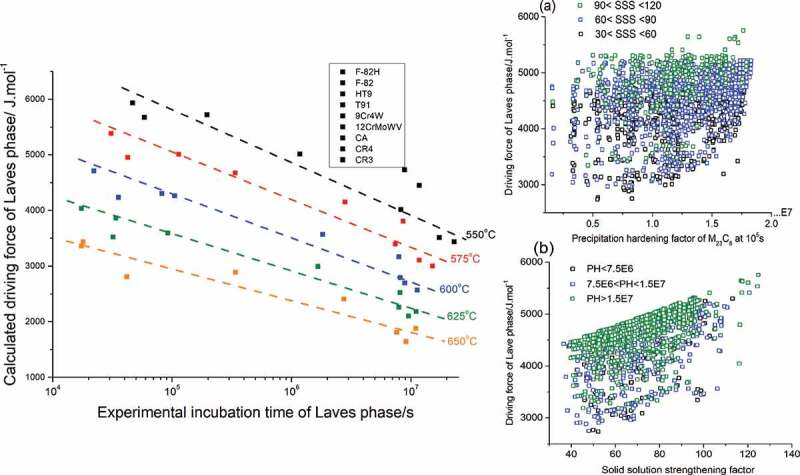

## 1. Introduction

Heat-resistant steels combining superior creep strength and great corrosion resistance at high temperature have been employed for decades in automotive, aerospace, fossil and nuclear power plants applications [1,2], but the industrial demand for materials with better performance for higher use temperature and longer expected lifetimes has led to ongoing research into the development of novel heat-resistant steels. Ferritic heat-resistant steels are attractive options because of their good thermal conductivity, low thermal expansion, and excellent cost efficiency [3,4]. Commercial ferritic heat-resistant steels are generally optimised by employing two strengthening mechanisms, precipitation strengthening by finely distributed particles (such as MX carbonitrides, where M stands for metal element and X is C or N [5,6] and NiAl/Ni_2_TiAl intemetallics [7]) and solid solution strengthening by solvent atoms (such as W, Mo and Al [8]) to achieve decent high temperature strength. The conventional alloy-design strategies are based on the so-called ‘damage prevention’ paradigm [9], i.e., the steel compositions and microstructures are tuned in such way that the formation of mechanical damage is postponed for as long as possible. But once the creep deformation-induced defects, such as creep cavities at grain boundaries, their dimensions will only increase until they merge into micro- and macrocracks, which ends the period of safe use of the installation.

The concept of self-healing has been proposed as an alternative method to enhance the lifetime of a wide range of materials [10–12]. In self-healing high temperature metals, mechanical damages can be repaired or mediated by local directional transport to the damage site of substitutionally dissolved chemical elements which dwell in a supersaturated state in matrix. The selection criteria to identify alloying elements, which can selectively precipitate at such grain boundary cavities in ferritic matrix to potentially provide a self-healing behaviour, have been discussed in detail by van Dijk and van der Zwaag [13]. Four substitutional alloying elements, i.e., Cu, Au, W and Mo, have been earmarked as potentially effective healing agents, and the healing of creep damage formed at 550°C in the corresponding Fe-X binary alloys has been studied in great depth [14–18]. Results showed that in Fe-Cu alloys, Cu-rich particles have a stronger preference to form within the grain interior, rather than in the creep cavities [19]. The ineffective consumption of the healing agents by precipitate formation at other locations than the creep cavities resulted in a poor healing efficiency for the Fe-Cu system. Physical arguments indicate that Au atoms have a stronger tendency to precipitate preferentially at free surfaces rather than in the grain interior. The high site-selectivity in combination with the high atomic mobility of Au resulted in a high efficiency for cavity-filling process in Fe-Au system [15,16,20–22]. While the finding was of great conceptual and scientific relevance, the use of Au as a useful healing agent for commercial creep resistant steels is most unrealistic because of its high price. However, in Fe-W and Fe-Mo alloys with Mo and W concentrations leading to 1 atomic percent supersaturation at the testing temperature, a similarly abundant precipitation of Laves phases in creep cavities was found to occur when experimental conditions were selected such that creep failure occurred at time scales up to 1 month [17]. Unintentionally, the experiments demonstrated that the healing agent should not only have a natural tendency to precipitate in the cavity, but also that the precipitation kinetics (nucleation and growth) should match the formation kinetics of the cavities in order to yield effective healing. It’s easily understandable that systems with a slow precipitation reaction may not show effective healing behaviour when the rate of damage accumulation is high enough. Similarly, in systems with very fast precipitation kinetics the precipitation may already take place before the grain boundary cavities are formed, and again no self-healing behaviour can be observed. In the present work we will introduce this kinetic aspect into our earlier genetic algorithm/thermodynamics computational design approach for the compositional optimisation of high performance creep resistant steels [23–27].

In the present work, W (via its contribution to the formation of Laves phase precipitates) is selected as the self-healing agent. A hybrid approach has been developed by considering various potential solutions as suggested by the Genetic algorithm selection and taking the Laves phase nucleation time parameter as a first-order indication for the desired kinetics of the healing reaction to find optimal compositions. The temperature-dependent compatibility between healing kinetics and mechanical properties is also addressed. Future work will aim to explicitly introduce the nucleation and growth kinetics of the defects, in order to account for the stress dependence of the creep damage evolution, as well as the corresponding healing reaction.

## 2. Model description: alloy by design

### 2.1. Model development

The design methodology used follows the ‘goal-means’ material design philosophy first proposed by Olson [28]. First the requirements imposed by the intended use conditions are translated into the corresponding mechanical and other properties, then the required properties are translated into required microstructure features. The desired microstructural features are converted to quantifiable criteria, which are finally linked to a specific composition and associated heat treatment conditions via physical metallurgy models with the guidance of thermodynamic principles. While the original model for the design of high strength oxidation-resistant creep steels only focussed on the required microstructure during the final use stage [29], it was soon realised that finding proper solutions requested setting quantified microstructural targets for every stage of the total thermal heat treatment [30]. The optimal composition best fulfilling the desired microstructural parameter targets can be found by any efficient optimisation method, but Genetic algorithms have been found to be quite effective and relatively robust for multi-parameter and multi-objective optimization. Genetic algorithms have an algorithmic structure inspired by evolutionary process in nature system by following the survival of the fittest principle. However, as the method is a statistical approach, the underlying implicit relationships reflecting the physical mechanisms remain hidden in the presentation of the set of potential solutions. In contrast, the conventional alloy-design method generally starts by educated guesses based on empirical relationships with physical or quasi-physical backgrounds, while the adjustments in composition are traceable in the changes of the properties. The obvious drawback is that in the case of a large number of input parameters and non-linear (inter-)relations, the conventional physics-based approach leads to a low search efficiency and no evidence that the true optimal solution has been identified. In this paper, the combination of these two approaches was made to construct a so-called hybrid model, in which the high efficiency in searching the composition range was integrated with the manual-selected compositional variation for unravelling the physical mechanisms of alloying elements. The model as developed for the optimisation of non-self-healing heat-resistant steels with ferritic, martensitic and austenitic matrix, and taking into account the kinetics of precipitate coarsening as well as the steady-state contribution of solid solution hardening has be described in detail in [23–27,31].

The composition ranges and the range of homogenisation temperatures from which the Genetic algorithm routine selected the candidate compositions and homogenisation temperature are given in Table 1. For each parameter 32 equally spaced values between the minimum and maximum value were set for optimization. The various imposed criteria in the program, be it constraints or optimisation parameters are described in more detail below. The model calls upon Thermo-Calc to calculate equilibrium fractions of phases present at the use temperature for each selected composition.

**Table 1.** Search range (in wt.%) for alloying elements and the homogenisation temperatures (in ^0^C).

	C	Cr	Mn	Si	W	Fe	T_homog_
Min.	0	8	0	0	0	Balance	800
Max.	0.1	20	5	5	3		1200

### 2.2. The microstructural parameters defined to reach the intended mechanical properties

The candidate steels are defined to have a fully ferritic matrix and to be strengthened by both precipitation strengthening and solid solution strengthening mechanisms. A typical heat treatment of ferritic steels includes an annealing treatment for achieving composition homogeneity, followed by a rapid cooling process to keep the ferritic matrix. To obtain a desirable final microstructure, candidate solutions should fulfil the following go/no-go criteria at the homogenisation temperature:

1. The equilibrium volume fraction of ferrite should be larger than 99%
2. The maximum level of primary carbides should be limited to 0.5% in vol.%

After homogenisation, the alloys are quenched to room temperature, and a nearly fully ferritic matrix can be maintained without additional phase transformation. However, during service the frozen high temperature state may decompose and new phases may form to satisfy thermodynamic equilibrium conditions. Ignoring for the time being the formation of the Laves phase to act as the healing agent, the following criteria are imposed to obtain the desired behaviour during service at desired temperature. The actual values selected by now are not unrealistic but remain arbitrary. The values affect the outcome of the optimal composition predictions but do not affect the functioning of the program as such.

1. The maximum amount for all undesirable phases (which leads to early component failure excluding M_23_C_6_ carbide and Laves phase) should be no more than 1 vol.%

To achieve a sufficient oxidation resistance at the exposure conditions:

1. A minimum chromium concentration of 8 wt.% should remain in solid solution in matrix to yield adequate corrosion and oxidation resistance.

For potential compositions selected by the Genetic algorithm meeting these four constraints, two strengthening factors, the precipitation hardening and the solid solution hardening, are calculated and then stored in a database. Precipitation hardening indicates the process that strengthens the matrix by producing uniformly dispersed particles in the microstructure. The occurrence of strengthening particles helps hinder motion of dislocations and thereby improves the strength of the material. The Precipitation hardening contribution (PH) in creep resistant steels is inversely proportional to the inter-particle spacing of the strengthening particles, which in general is a function of the particle volume fraction, the initial particle size and coarsening kinetics of the precipitates [23,26,31]. The following expression is used to calculate the time-dependent precipitate strengthening factor while taking into account precipitate coarsening and its temperature dependence:

(1) $$\sigma_{p}\propto 1\;/\;L=\sqrt{f_{p}}\;/\;r_{t}=\sqrt{f_{p}}\;/\;\sqrt[{3}]{r_{o}^{3}+Kt}$$

(2) $$r_{0=2\gamma \;/\;\Delta G_{V}}$$

(3) K=8γVmp∑i=1n9(xip−ximp)2ximpDi ximpRT

where L is the average inter-particle spacing, f_p_ is the equilibrium volume fraction of the strengthening precipitates at the service temperature, r_t_ is the particle size at the service time t, r_0_ is the critical precipitate nucleus size being set the initial size of the particle, γ is matrix-precipitate interfacial energy, ΔG_v_ is volume thermodynamic driving force for the precipitation. V_m_^p^ is the molar volume of precipitate. K is the factor of coarsening rate and t is the exposure time at the high temperature. $x_{i}^{p}$ and $x_{i}^{mp}$are the equilibrium mole fraction of the precipitation forming elements on the matrix and precipitate sides of their common interface respectively. T is the service temperature and D_i_ is the corresponding diffusion coefficient. In the calculations the interfacial energy is arbitrarily set at a fixed value of 0.5 J/m^2^ [32] irrespective of the precipitate size or type. This is a slight simplification but helps in illustrating the effect of precipitate coarsening. All thermodynamic parameter values including f_p_, ΔG_v_, x_i_^p^, x_i_^mp^, D_i_ and V_m_^p^ required during the calculations are calculated via Thermo-Calc using the TCFE9 and MOBFE1 databases. The equation shows that the highest strengthening factors are obtained for high volume fractions and small and barely coarsening precipitates. The computational details of the model and its application to MX precipitation strengthened creep resistant steel design can be found elsewhere [23,26,31].

Solid solution strengthening works by adding atoms of alloying element to the crystalline lattice of the base metal, forming a solid solution. The local nonuniformity in the lattice due to the alloying element makes plastic deformation more difficult by impeding dislocation motion through stress fields, and thereby improves the yield strength of the material. The amount of solid solution strengthening depends on both the chemical nature of the solute atom and the maximum concentration which can be brought into solid solution. The solid solution strengthening factor (SSS) was then taken to be the weighted contribution of atomic concentration of solutes, which can be formulated as

(4) $$\Delta \sigma_{ss}=\alpha_{T}\underset{i}{\sum }C_{i}x_{i}^{m}$$

Where α_T_ is a temperature-dependent scalar, C_i_ is the (room temperature) solid solution strengthening coefficient which combines the effects of size misfit and modulus misfit, and $x_{i}^{m}$presents the equilibrium mole fraction of the elements dissolved in matrix. For the solid solution elements in this work, the strengthening coefficients at room temperature were determined from literature data on binary Fe-M systems and values used are reported in Table 2. The value of the parameter α_T,_ which takes into account the temperature dependence of solid solution hardening was determined by a quantitative analysis [25] of the experimentally determined minimum yield strength values at elevated temperature [33] as well as the thermodynamically calculated dissolved element concentration. A value of α_550_ = 0.98 resulted from such an analysis.

**Table 2.** Room temperature strengthening coefficient for alloying elements dissolved in ferrite (MPa per at. %) [34,35].

Element	C	Si	Mn	Mo	W	Cr
C_i_	1103.45	25.80	16.90	15.90	31.8	2.60

### 2.3. The microstructural parameters for healing properties

With the constraints for requested microstructural features being present, as well as the quantitative factors to evaluate the strengthening mechanism being imposed, solutions with decent mechanical properties can be identified and ranked. Apart from the considerations of strength, the self-healing properties need to be specifically specified in our design routine as a new property-evaluation domain. From a microstructural perspective, the amount of healing product to be formed should be equal or slightly higher than the total damage volume, in order to provide sufficient filling capability. The most extensive data sets for nano-tomographic data for creep damage and healing currently available [16] indicate that the total volume fraction of creep cavities before failure varied between 0.11 and 0.76 vol.% for the Fe-Au model system. In the model the healing product volume criterion was set as follows

1. The volume fraction of Laves phase which can form at the grain boundary cavities should be higher than 1% to sufficiently fill the total volume of cavities.

**Figure 1.**
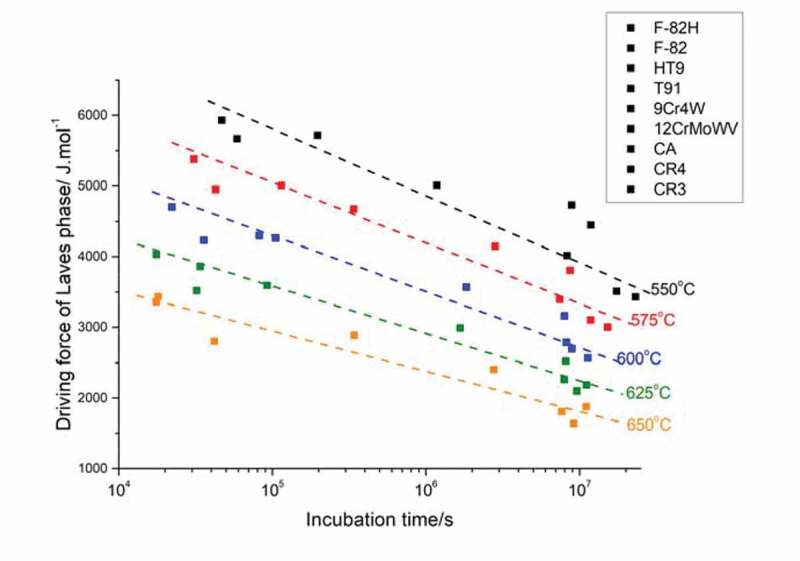
Experimentally observed incubation times for Laves phase precipitation at several temperatures as reported for nine commercial steels [39–42].

The key problem then becomes the definition of the evaluation factor for self-healing properties. To achieve the effective healing reaction, it is crucial that the nucleation time for such Laves phase precipitates coincides more or less with the time at which creep cavities start to form or reach a healable size. To simplify the problem and circumventing the formation kinetics of cavities, the incubation time for Laves phase precipitates to form was set a decision parameter, while in this sense the incubation time is regarded more as a constraint than as an optimisation parameter. Reliably predicting the formation kinetics of Laves phase precipitates based on physical metallurgy principles seems impossible, as classic precipitate nucleation theories [36–38] for simple metallic systems with few alloying elements were considered to be inappropriate for the multi-component steels considered here. In this work, we resorted to establishing an empirical relation based on reported incubation times. To this aim, reported incubation times for Laves formation in nine commercial W high temperature steels have been collected and the thermodynamic Driving force (DF) for its formation at the appropriate temperature has been calculated. The data are shown in Figure 1.

After grouping the data by temperature, a rather linear relationship between the thermodynamic driving force and the logarithm of the incubation time was obtained. The formation behaviour of healing agents can be thus quantitatively indicated by the new optimization domain obtained from simulated results. So, once the creep temperature and the desired onset time have been selected, the corresponding driving force can be determined, which can be employed as a constraint in the composition optimisation program. It should be pointed out that the incubation times reported are those for Laves phase precipitation in the bulk of the material which will more or less slower than that for precipitation on a free (internal cavity) surface but with suitable models converting nucleation barriers for bulk precipitation to that for surface precipitation, more accurate estimates of the incubation times can be made.

## 3. Results

### 3.1. The composition distribution of all solutions

**Figure 2.**
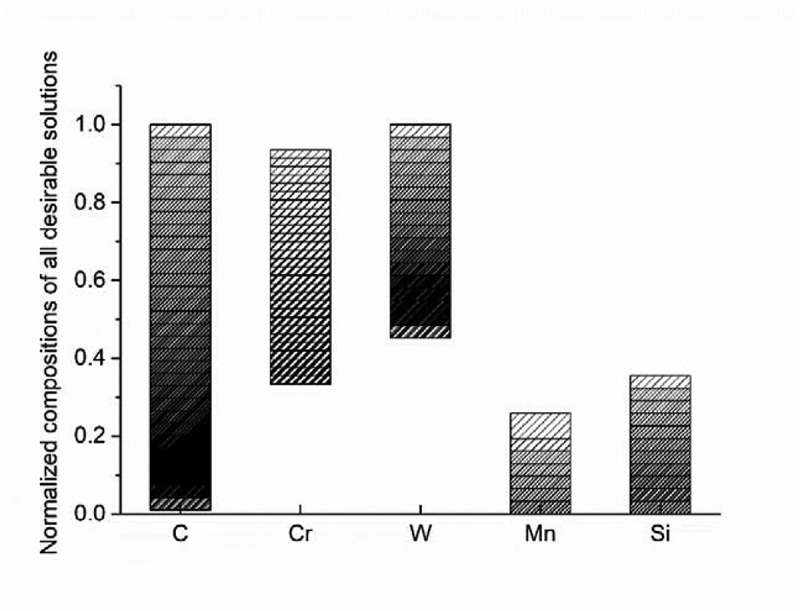
The composition distribution of all potentially feasible compositions normalized to the total composition search range in Table 1. The density of column patterns represents the number of solutions meeting the criteria.

The composition distribution of all potential solutions identified by the generic model is presented in Figure 2, with their compositions normalized to the composition search range shown in Table 1. The density of column patterns indicates the number of solutions meeting the criteria set. As shown in this figure, C can occupy the whole composition search range, since the upper limit of C in Table 1 was set to a relatively low value. it is interesting to point out that more identified solutions have a low C level, as indicated by the pattern density peaking at around 0.2 (0.02% C). The reason for this mainly resides in the microstructural requirements to guarantee a ferritic matrix. The Cr concentration is nicely restricted and evenly distributed over a medium range. This is due to the fact that a low Cr content (<10%) will violate the go/no-go criteria for corrosion resistance, while an excess of high Cr (>18%) will lead to the extensive formation of undesirable σ phases. The more common W concentrations are located around a relatively high level (>1.36%), owing to the relatively high amount of Laves phase to form in the creep cavities. The distribution of Mn levels peaks at a low range (<1.29%) since Mn stabilizes austenite at high temperature, which leads to violation of the required fully ferritic matrix. The Si levels in the identified potential solutions also remains at a relatively low level (<1.77%) as Si promotes the formation of the unwanted σ phase

### 3.2. The mechanical properties at 550°C

**Figure 3.**
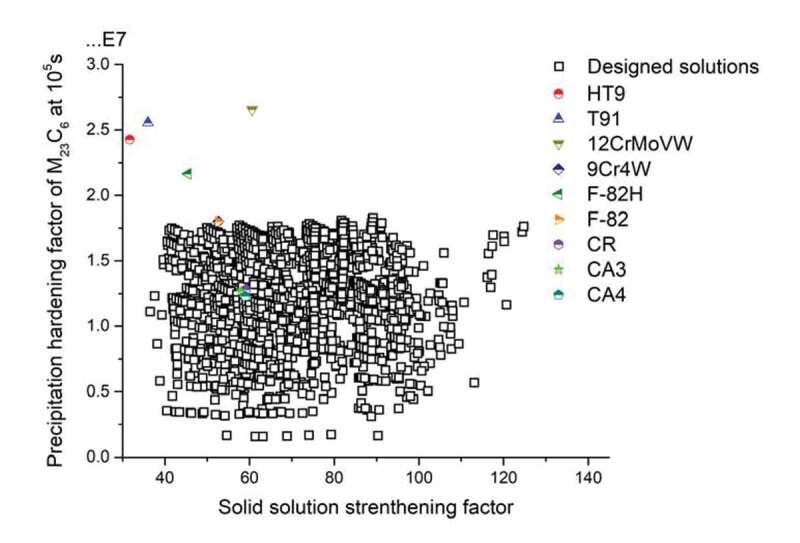
PH factor of M_23_C_6_ carbides after 10^5^s at 550°C versus SSS factor of those solutions meeting all go/no-go criteria. The values of existing commercial steels, indicated by star symbols, are also put on the plot to compare to the identified possible solutions to the current state of the art steels.

Abiding by the design route, the Genetic algorithm-based search covered about 10^9^ unacceptable or less well-performing variants and finally identified around 10^4^ desirable solutions. All candidate solutions which fulfil all the go/no-go constrains are recorded for further analysis, with the Precipitation hardening factors of M_23_C_6_ carbides at the time of 10^5^s versus Solid solution strengthening factors shown in Figure 3. The solutions with the combination of high PH and SSS values which are expected to possess the best mechanical properties, are located in the right top corner of the figure. To benchmark the currently proposed potential solutions, the calculated hardening parameter for existing commercial creep steels are plotted in the figure too, with their PH factors as well as the SSS factors indicated by stars. Compared to the existing steels, the designed solutions are relatively weak in precipitation strengthening contribution; the best-performed solutions can reach 2/3 value of PH factor of commercial grade 12CrMoWV (marked as yellow triangle in Figure 3). However, given the fact that we focussed on M_23_C_6_ precipitates and did not include MX precipitation optimisation, and the fact that the we currently deal with five-element systems only compared to commercial steels which have up to 11 alloying elements, the difference in precipitation strengthening is deemed acceptable. Besides, it is worth noting that in terms of SSS factor, wide range of the solutions can reach the values that are beyond the performance of existing alloys. The simulation in mechanical properties shows that at the service temperature 550°C, the designed solutions can reach the comparable level in strength compared with the existing steels.

### 3.3. The design results with self-healing properties

**Figure 4.**
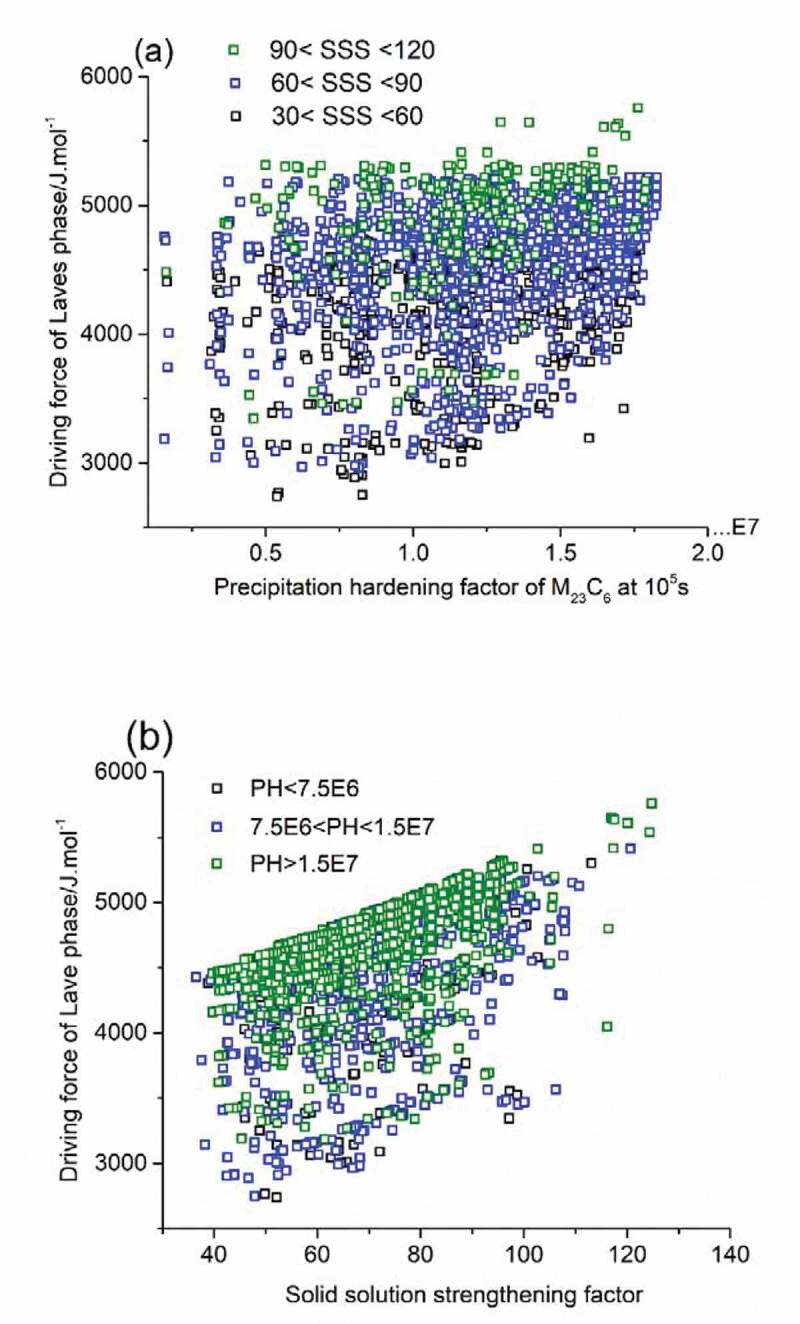
PH factors of M_23_C_6_ precipitates as strengthening particles at 10^5^s (a) and the SSS factors (b) versus driving force of Laves phase as self-healing agents meeting all go/no-go criteria at a temperature of 550°C.

Applying the linear relationship between calculated driving force for Laves phase formation and experimental incubation time for precipitation, and taking the driving force for Laves phase formation as a quantifying parameter for the timely occurrence of self-healing, the properties of acceptable solutions in Figure 3 are redrawn and shown in Figure 4 with new criteria domain. In Figure 4(a), precipitation hardening factor was set as the key optimization parameter for the mechanical property contribution, but the colour coding of the dots indicates their individual SSS level. It is found that black dots (solutions with SSS values between 30 and 60 MPa) and blue dots (solutions with SSS values between 60 and 90 MPa) are distributed evenly within the whole PH/DF domain while solutions with a high SSS value, i.e. the green dots, tend to crowd in the high driving force value domain. The best compositions, i.e. having a combination of a high value for the PH and SSS factor as well as a low driving force of Laves phase formation are indicated by the green-coloured dots located at the bottom right corner of the figure. Similar method was applied to obtain Figure 4(b), with SSS value being the main optimization parameter versus Laves driving force. In this figure, the density of the dots is obviously higher in the high driving force range, while the dots located in the relatively low driving force domain are sparser.

The validation of PH factor and SSS factor as criteria for mechanical properties have been confirmed by our formal work, see reference [23,26]. For the newly defined parameter, driving force of Laves phase, dedicated experimental tests are necessary and in preparation to validate the applicability as a criterion to tune the occurrence of self-healing at either short times (i.e. for high creep stress levels) or to intermediate (i.e. for intermediate creep stress levels) or to very long times (i.e. for lower creep stress levels). In the following section, representative solutions are presented and it is shown that, while keeping the predicted mechanical properties constant, the time at which healing is to be activated affects the recommended optimal composition.

### 3.4. Example alloys with the effect of planned healing initiation time on their compositions

The complete preliminary solution pool is shown in Figure 4(a and b), with all properties of the solutions being stored digitally. Then, a classification of the solutions is made manually with the help of a conventional composition optimization method, while the effects of variation in chemical concentration on the targeted properties are explored. The selected example alloys No.1, 2, 3 (see Table 3) have a similar level of PH factor value (~8.6 × 10^6^) and SSS factor value (~ 75), but have widely different values for the Laves phase driving force 2964 J/mol, 4218 J/mol and 4848 J/mol respectively, corresponding to healing initiation times of 1000 days, 75 days and 10 days. While the estimated healing initiation times are to be regarded as crude first-order estimates, the data point to be an important issue of proper healing initiation, which by now have not been presented in the literature yet.

**Table 3.** Compositions of three selected alloys (in wt.%) and their PH factor, SSS factor driving force for Laves phase formation and estimated healing initiation times at 550°C.

	*C*	*Cr*	*Mn*	*Si*	*W*	*T_homog_/^0^C*	*PH@10^5^s M_23_C_6_*	*SSS*	*DF of Laves/J.mol^−1^*	*t_init_* */days*
1	0.026	12	0.81	0.32	1.45	1200	8.65 · 10^6^	69	2964.2	~ 1000
2	0.023	12	0.32	0.65	2.23	1200	8.55 · 10^6^	75	4218.4	~ 75
3	0.023	13.03	0.01	0.97	3.0	1200	8.71 · 10^6^	80	4848.8	~ 10

The three alloys are selected to yield widely different healing kinetics as shown in Table 3 but comparable yield strength values. Alloy 1 is expected to heal the damage if the experimental loading conditions are set such that damage and failure will occur on a time scale of about 1000 days. Alloy 3 should show effective healing in case the load level is set to a value such that damage will start to form after 10 days service. The required difference in initiation times for Laves phase formation has interesting yet logical effects on the recommended compositions. With the Laves phase driving force increasing from alloy 1 to 3, the concentration of Mn decreases, the Si and W alloying levels goes up, while the C and Cr concentration are almost unchanged. The experimental investigation from Hosoi [42] confirms that the addition of Si and a decrease in Mn level will promote the formation kinetics of Laves phase, which agrees well with our prediction results. The increase in alloying level of W as the Laves forming element leads to the increase in laves driving force, which is also predictable given the higher level of supersaturation.

### 3.5. The effect of higher use temperatures on the possibility to combine well-timed healing with decent mechanical properties

The discussion so far focussed on a use temperature of 550°C, where precipitation strengthening plays an important role in for providing the mechanical properties. However, there is a strong incentive in the industry to raise operating temperatures in power stations and other thermal installations. Therefore, in the following content, we attempt to broaden the application window to a higher temperature domain, by setting the temperature at 600°C or 650°C.

**Figure 5.**
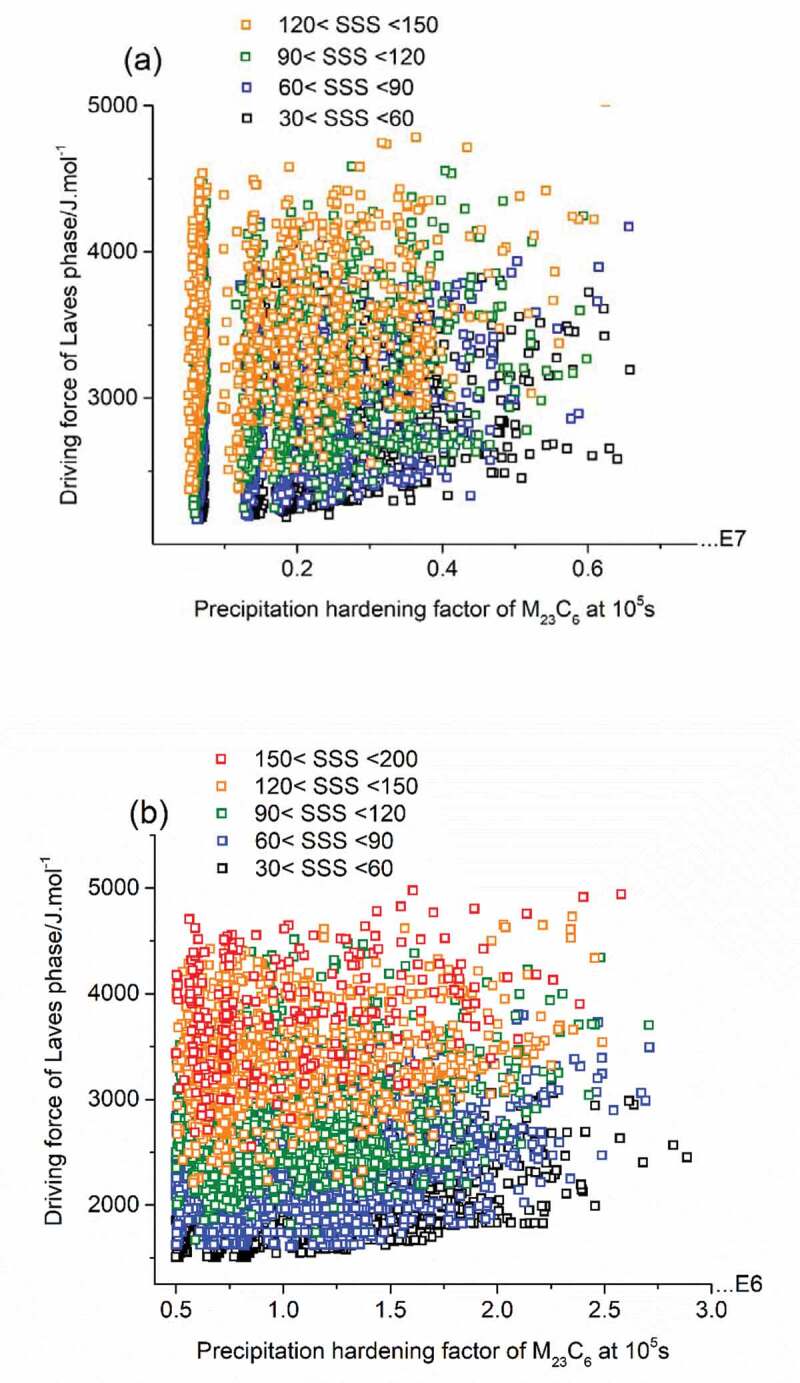
PH factors of M_23_C_6_ precipitates as strengthening particles at 10^5^s versus driving force of Laves phase as self-healing agents meeting all go/no-go criteria at the temperature of 600°C (a) and 650°C (b).

Following the same alloy-by-design concept discussed in previous section, similar results have been obtained which are plotted in in Figure 5(a and b) for 600°C and 650°C respectively. For a use temperature of 600°C, as in Figure 5(a), the minimum value of driving force of Laves phase was 2170 J/mol (corresponding to a healing initiation time of 500 days), located in the bottom left corner of the image. By comparing the data in Figure 5(a) to that in Figure 4(a) it is clear that precipitation hardening factor at 600°C was halved compared to that at 550°C. The degradation of particle-strengthening contribution as temperature increases is predictably due to the faster coarsening rates of precipitates brought by the higher atomic mobility at higher temperature. At 650°C the precipitation hardening factor drops even more sharply indicating that M_23_C_6_ precipitation hardening does not contribute to the strength anymore. The minimal Laves phase driving force drops to a value of 1500 J/mol, which would correspond to a maximal time for precipitation initiation of approximately 40 days.

**Figure 6.**
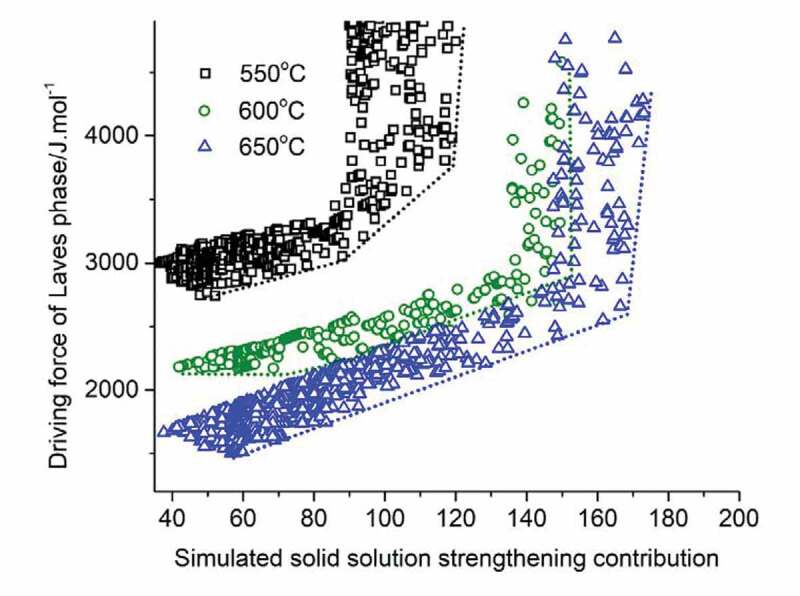
Properties values for SSS factor and Laves phase driving force on the Pareto front at different temperatures.

With the contribution of precipitate hardening becoming minor, it is important to shift the focus to the contribution of solid solution hardening. While the α_T_ value decreases with temperature (α_600_ = 0.64, and α_650_ = 0.51, obtained by the method from [31]), the total SSS contribution can be higher as the solubility of most elements increase with increasing temperature. Thus, it would be of interest to investigate the compatibility of a low Laves driving force and a high solid solution strengthening as a function of the temperature. To this aim, a Pareto front plot was constructed between the solid solution strengthening factor and the driving force for Laves phase precipitation, with data grouped according to the use temperature of 550, 600 and 650°C. The data are shown in Figure 6.

The figure shows that as the temperature increases, the maximum (relative) contributions that solid solution strengthening can provide increases, which means higher strength can be obtained by employing solid solution strengthening mechanism at elevated temperature. But at higher solid solution levels, the driving force for Laves phases to precipitate becomes higher simultaneously. Under these conditions, precipitate formation will take prematurely, i.e., before there is serious creep damage. Taking an arbitrary time of 10^7^ seconds (115 days) as a suitable time to initiate healing, the figure shows that a combination of decent solid solution and relatively late initiation of healing due to Laves phase precipitation in cavities can only be achieved for use temperatures in the range 550–600°C, but that at 650°C such a combination of desirable properties is predicted not to exist.

The outcome of the above analysis clearly depends on the precision of the precipitate nucleation kinetics (either in the bulk or on internal free surfaces). And other experimental data could have led to quantitative different but qualitatively the same conclusions. Hence, the analysis presented here proves that the kinetics of grain boundary precipitation and that of the formation of the precipitates in the cavities should be included explicitly in future self-healing alloy design models as it greatly influences optimal compositions.

## 4. Conclusions

1. A hybrid model has been developed by combing the generic alloy-by-design model for W-containing non-self-healing ferritic steel grades with new criteria for the self-healing reactions and in particular the onset time for the filling of the grain boundary pores by Laves phase precipitates. The high efficiency in the pre-screening of the multi-element composition range was integrated with an operator driven analysis of the effect of parameters.
2. Self-healing ferritic steels with predicted decent strength values to be operated at 550°C by combining precipitation strengthening and solid solution strengthening. To quantify the self-healing properties, an empirical relationship is derived which captures the experimentally observed incubation time for Laves phase formation in existing commercial alloys and links these conditions to a chemical driving force for Laves phase formation.
3. It is demonstrated that the time at which the autonomous healing reaction is to be initiated strongly influences the optimal compositions of the steels.
4. The current alloy design analysis indicates that at temperatures above 600 °C it might be hard to obtain steels with combine good mechanical properties and a long-term self-healing behaviour.
